# Properties and Applications of Polyvinyl Alcohol, Halloysite Nanotubes and Their Nanocomposites

**DOI:** 10.3390/molecules201219884

**Published:** 2015-12-19

**Authors:** Tayser Sumer Gaaz, Abu Bakar Sulong, Majid Niaz Akhtar, Abdul Amir H. Kadhum, Abu Bakar Mohamad, Ahmed A. Al-Amiery

**Affiliations:** 1 Department of Mechanical & Materials Engineering, Faculty of Engineering & Built Environment, Universiti Kebangsaan Malaysia, Bangi, Selangor 43600, Malaysia; majidniazakhtar@ciitlahore.edu.pk; 2 Department of Machinery Equipment Engineering Techniques, Technical College Al-Musaib, Al-Furat Al-Awsat Technical University, Al-Musaib, Babil 51009, Iraq; 3 Department of Physics, COMSATS Institute of Information Technology, Lahore 54000, Pakistan; 4 Department of chemical & Process Engineering, Faculty of Engineering & Built Environment, Universiti Kebangsaan Malaysia, Bangi, Selangor 43600, Malaysia; amir@eng.ukm.my (A.A.H.K.); drab@eng.ukm.my (A.B.M.); dr.ahmed1975@gmail.com (A.A.A.A.); 5 Fuel Cell Institute, University Kebangsaan Malaysia (UKM), Bangi, Selangor 43000, Malaysia

**Keywords:** polyvinyl alcohol, halloysite nanotubes, properties, biomedical application, biocompatibility

## Abstract

The aim of this review was to analyze/investigate the synthesis, properties, and applications of polyvinyl alcohol–halloysite nanotubes (PVA–HNT), and their nanocomposites. Different polymers with versatile properties are attractive because of their introduction and potential uses in many fields. Synthetic polymers, such as PVA, natural polymers like alginate, starch, chitosan, or any material with these components have prominent status as important and degradable materials with biocompatibility properties. These materials have been developed in the 1980s and are remarkable because of their recyclability and consideration of the natural continuation of their physical and chemical properties. The fabrication of PVA–HNT nanocomposites can be a potential way to address some of PVA’s limitations. Such nanocomposites have excellent mechanical properties and thermal stability. PVA–HNT nanocomposites have been reported earlier, but without proper HNT individualization and PVA modifications. The properties of PVA–HNT for medicinal and biomedical use are attracting an increasing amount of attention for medical applications, such as wound dressings, drug delivery, targeted-tissue transportation systems, and soft biomaterial implants. The demand for alternative polymeric medical devices has also increased substantially around the world. This paper reviews individualized HNT addition along with crosslinking of PVA for various biomedical applications that have been previously reported in literature, thereby showing the attainability, modification of characteristics, and goals underlying the blending process with PVA.

## 1. Introduction

Polyvinyl alcohol (PVA), which is essentially made from polyvinyl acetate through hydrolysis, is easily degradable by biological organisms and in water is a solubilized crystalline structure polymer [1]. PVA is an artificial polymer that has been used during the first half of the 20th century worldwide. It has been applied in the industrial, commercial, medical, and food sectors and has been used to produce many end products, such as lacquers, resins, surgical threads, and food packaging materials that are often in contact with food [2]. PVA is a biodegradable imitation of natural polymers used in paper coating and textile sizing [3]. This polymer is widely used by blending with other polymer compounds, such as biopolymers and other polymers with hydrophilic properties; it is utilized for various industrial applications to enhance the mechanical properties of films because of its compatible structure and hydrophilic properties [4]. Some man-made polymers, which are made from non-renewable and non-biodegradable sources, such as PVA, are available [5]. Polymers that are biologically decomposable originate from petroleum-based synthetic materials, which decompose naturally under aerobic (composting) or anaerobic (landfill) conditions [6,7]. PVA is a widely used thermoplastic polymer that is benign to living tissues, harmless, and nontoxic. This polymer is widely investigated because of its use in cross-linked products and nanofillers [6,8,9]. PVA is a biodegradable polymer, and its degradability is enhanced through hydrolysis because of the presence of hydroxyl groups on the carbon atoms. Moreover, it is water-soluble and has a hydrophilic nature [6,10,11,12,13,14]. Rates and environmental conditions for degradation may vary for many polymers, such as PVA [6,15,16,17,18]; these conditions include composting in the presence of oxygen, underneath soil layers, in aqueous media, and even in anaerobic circumstances. In the paper industry, several synthetic, copolymers, and conventional polymers, such as polyurethane (PU), polystyrene, and maleic anhydride SBR, SMA, polyacrylamide PAM, and PVA, are typically utilized to enhance the features and characteristics of paper by coating a film layer onto the whole sheet [7,19]. PVA is a major artificial polymer that has been available for more than nine decades. PVA is synthesized through the saponification process of poly(vinyl acetate) [20,21] and has long been used by blending with other natural polymers because of its film-forming features [22,23]. The complete dissolution of PVA in water is bound by its intrinsic properties, which require the water temperature to be at ~100 °C with a holding time of 30 min [23]. All PVA grades are hydrophilic and depend on certain factors, such as molecular weight, element dimensions of distribution, and particle crystal structure [23,24]. The resulting gel properties have been reported in literature [25]. 

Natural deposits of aluminosilicate (Al_2_Si_2_O_5_(OH)_4_∙nH_2_O) are chemically similar to kaolin, but with halloysite nanotubes (HNTs), *i.e.*, hollow tube-shaped, micro- to submicrosized structures have limited high aspect ratio. HNTs are quarried naturally from many countries around the world, such as Japan, China, America, South Korea, Brazil, France, and Turkey [26]. Composite films with enhanced mechanical and thermal properties can be prepared by casting from a PVA–HNT solution. The enhancement of the polymer properties is accredited to the uniform dispersion of the nanotubes within the polymer matrix, which results in enhanced interfacial interactions in the composite systems. Recently, the role of HNTs as a viable and biocompatible candidate for biomaterial applications has been verified [27]. HNT toxicity has been studied, where the course of cell absorbance of HNTs is conceived in various cells [28]. The main aim of this article is to discuss the synthesis, properties, and applications of PVA, HNTs, and their PVA–HNT nanocomposites. The individualized HNT addition and crosslinking of PVA for various biomedical applications are also reviewed, thereby showing the prospective accessibility, modification of characteristics, and goals underlying the blending process with PVA.

## 2. Chemical Structure of PVA

**Figure 1:**
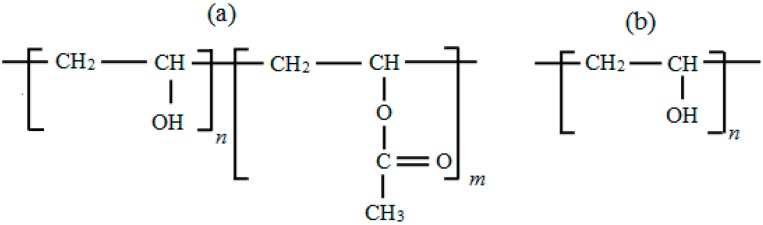
Structural formula for PVA: (**a**) partially hydrolyzed; (**b**) fully hydrolyzed [2].

The properties of polyvinyl acetate depend on the extent or degree of its hydrolysis, specifically whether it is full or partial (Figure 1), which in turn dictates its categorization into two groups, namely, (a) partially hydrolyzed and (b) fully hydrolyzed.

**Figure 2:**
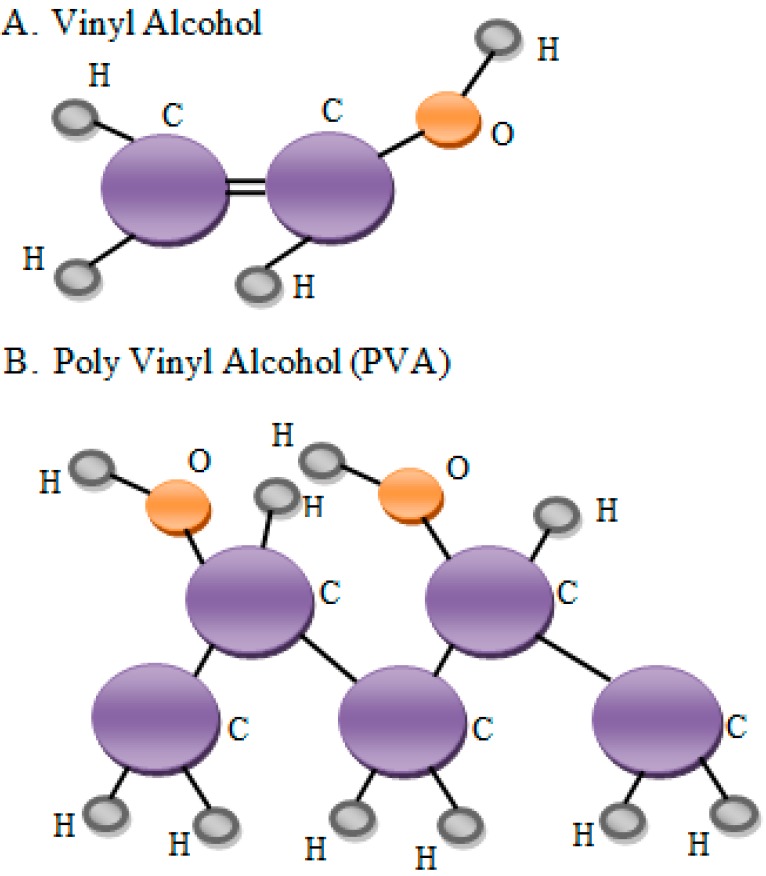
(**a**) The structure of vinyl alcohol; (**b**) PVA is synthesized by the hydrolysis of polyvinyl acetate [26].

The molecular weights obtained for PVA products may vary (20,000–400,000), depending on the length of the initial vinyl acetate polymer, the level of hydrolysis to eliminate the acetate groups and whether it occurs under alkaline or acidic conditions [2]. Figure 2 shows the structure of PVA. Hydrolysis levels vary from what is considered a typical value of 80% to reach more than 99%. Nearly fully hydrolyzed forms result in forming PVA hydrogels with tuneable properties through crosslinking of the linear polymers, which subsequently result in polymer (gel)-fluid (sol) species. Polymer contents affect the physical status of the resulting material: low polymer content results in a soft materials because the fluid moves freely through the matrix, whereas a higher polymer content results in considerable stiffening and strengthening of the material’s matrix [29]. Studies on the diffusive permeability of solutes in PVA gel membranes and the application for separation have been delineated because of chemical stability, particularly on film-forming, and hydrophobicity [30].

## 3. Chemical and Physical Properties of PVA

The chemical and physical properties of PVA may vary based on the percentage of hydrolysis, which determines the PVA grade and its molecular weight [2]. The surface properties of PVA fillers are fundamentally significant in the selection criteria of PVA fillers [3]. PVA itself has substantial tensile strength, more flexibility, and hardness and gas and aroma barrier characteristics. Compared with any other known polymer, PVA demonstrates remarkably superior features as an oxygen barrier; however, to avoid the degradation of its permeability toward gas, it must be protected from moisture [4]. PVA, like proteins, is a water-soluble polymer. The water solubility and physical properties of PVA, including its film form, are highly affected by the degree of hydrolysis, molecular weight, and its crystal precipitation [5]. PVA is partially crystalline upon formation and is characterized by properties such as chemical resistance, water solubility, and biodegradability. The similarity in physical properties makes it compatible with human tissues. Biocompatible PVA has a structure that can absorb protein molecules and engage with minimal cell adhesion and has no toxic effects, therefore, PVA membranes have been widely developed for biomedical applications [30]. PVA can chemically bound to or physically entangled with a nanoparticle surface [31]. PVAs are very common polymers, widely used as surface materials, which should be retained on water surface [32,33,34], in a huge range of fields as films [35,36] and glues [37] because of their exceptional chemical and physical properties [38,39,40], biocompatibility, stability to temperature variation, and non-toxicity [41,42].

## 4. Chemical Structure of HNTs

**Figure 3:**
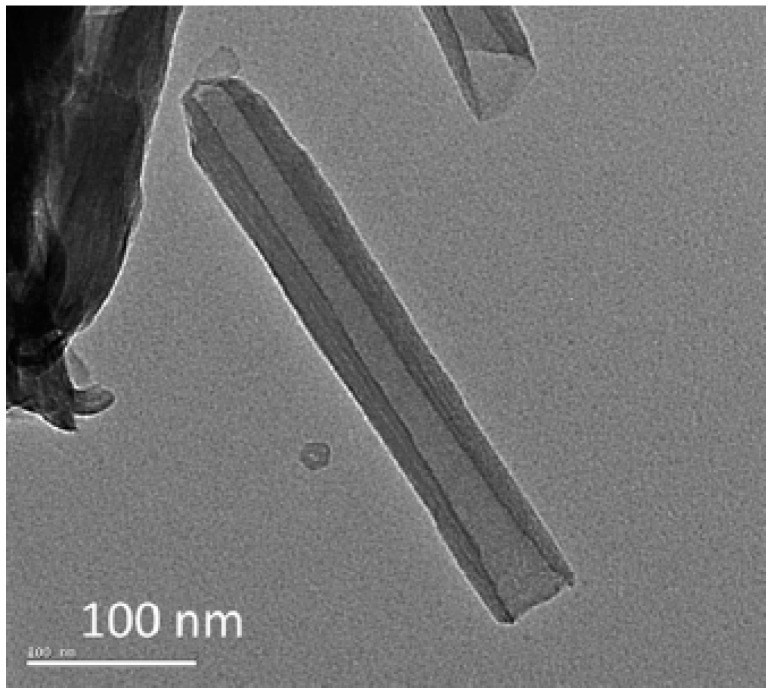
TEM micrograph of HNTs [44].

Micrographs of HNTs can be seen in Figure 3. A predominant percentage of the halloysites are of tube-shaped structures have the following measurements: the length varies from 150 nm to 2 μm, the outer diameter ranges from 20 nm to 100 nm; and the lumen diameter ranges from 5 nm to 30 nm. Given the distinctive and differential morphological aspects of HNTs, uncommon charge distributions, surfaces with lower hydroxyl density, and crystals with an unequalled structure are observed [7]. The featured characteristics of PVA–HNTs films were determined through the tensile strength, optical transmittance, and scanning electron microscopy [43].

**Figure 4:**
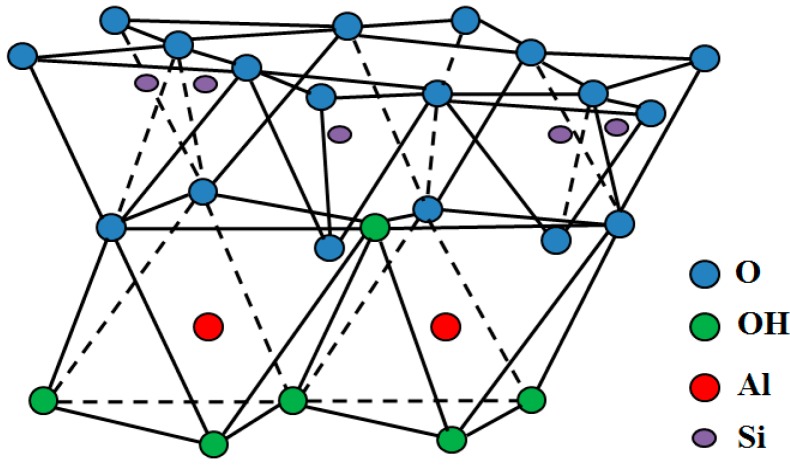
Crystalline structure of HNTs [46].

Figure 4 illustrates a crystalline-shaped typical unit of HNTs, which comprises a bilayer formation and two kinds of –OH groups, as follows: (a) the outer –OH groups located in the unshared plane of the tetrahedral (silicon and oxygen) sheet and (b) the inner hydroxyl groups located in the shared octahedral (aluminum and oxygen) sheet. Consequently, siloxanes form the outer side of HNTs, with a small amount of silicon hydroxyl groups positioned in the HNTs’ endings and surface defects. However, most of the aluminum hydroxide groups are located on the inner side. The development of H-bonding affects the blue shift of the Fourier transform infrared (FTIR) absorption of Si-O stretching because most of the aluminum hydroxide pairs are placed on the inner side of the crystalline formation, and the blue shift of luminol is far less than that for the Si-O group [45].

## 5. Advantages and Disadvantages of PVA and HNTs

PVA has been the subject of intensive research because it has many applications in industry. Under wet conditions, its properties are diminished because of the plasticizing action of water molecules [47]. HNTs more likely reduce the PVA decomposition to a considerable extent, but they are not efficacious in ameliorating the abstraction of the linked groups. The combination procedure at lower HNT concentrations is more effective in the thermal decomposition of composite films compared with the higher HNT concentration cases. Its abundance as a low-cost polymer is rendering it more attractive than other polymers [48,49]. Physical blending and chemical modification by grafting, interpenetrating polymer networks (Table 1), and crosslinking method greatly contribute to the alleviation of such disadvantages [50]. 

**Table 1:** Summary of main properties.

No.	HNTs	PVA
1	Natural, nontoxic [51,52]	Non-toxic [48,49]
2	Non-swelling [51,52]	Hydrogels exhibit swelling feature in water with the peculiar characteristics of retaining water within its matrix without dissolving [50].
3	Compatibility polymers oriented, such as polypropylene and polyethylene [51,52]	PVA is more biologically compatible [48,49]
4	Excellent mechanical properties such as tensile strength with 5 wt % HNTs increase 300% and thermal stability because HNTs are stable even at very high temperatures [3,53] in PVA film.	PVA has relatively low strength and thermal stability for some applications, excellent mechanical properties such as: strength (1.6 ± 0.1 GPa), elastic modulus (48 ± 3 GPa), strain (6.5% ± 1.4%), and toughness (40 ± 6 J·g^−1^) [54] and flexibility in dry state [47]
5	Formula [Al_2_Si_2_O_5_(OH)4∙nH_2_O] [55]	Formula [-CH_2_CHOH-]_n_
6	HNT is naturally occurring, will also have the benefit to reinforce PVA and impart other biological properties to the bionanocomposites, such as drug/gene delivery capacity without fear of being carcinogenic [53]	To overcome the limited biological performance and to enhance the mechanical properties of PVA, a new class of engineering designed PVA bionanocomposites has been introduced recently [53]

## 6. Applications of PVA and HNTs

For over 50 years, hydrogels have been invented and applied in numerous biomedical disciplines, such as production of contact lenses and absorbable sutures, osteoporosis, asthma treatment, and neoplasms. Thus, much attention has been given to the use or modification of different polymeric materials that can be currently used for biomedical devices to fulfill the increased need for those materials in medical applications.

### 6.1. Biomedical Applications of PVA and HNTs

PVA is already used in biomedical applications for its compatibility [25]. PVA composites, such as PVA gels, are used in different biomedical fields, such as in the manufacturing of contact lenses, artificial heart surgery, drug delivery systems, and wound dressings. In medical devices, PVA is used as a biomaterial because of its highly favorable properties, such as biocompatibility, nontoxicity, non-carcinogenic, swelling properties, and bioadhesive characteristics. This material is very useful and desirable for biomedical application and uses. Table 2 identifies some non-implant and implant devices that are currently made of different PVA forms and HNTs [26].

**Table 2:** Uses of PVA and HNTs in non-implant and implantable devices [26,52].

Device Type	Product PVA	Product HNTs
Non-implant devices	Surgical sponges and packing	Diuretic drug transportation to remove hazardous species
	Eye wetting drops	Sustained release of drugs, food additives, and fragrances
	Contact lenses	Antimicrobial agents
Implantable devices	Hydrophilic coatings (Catheters, leads, *etc.*)	Human breast cells
	Vascular embolic agents	Fibroblasts
	Tissue adhesion barriers	Corrosion protection implant alloys
	Nerve guides	Biosensors
	Cartilage replacements	Used in advanced ceramic materials, especially biocompatible implants

Crystalline structures can be controlled by modifying the chemical composition of OH groups [32]. Bio-inertness and compatibility are other PVA properties that have implications in advanced medical fields, hemodialysis, drug delivery system, and implantable medical devices [38,56]. PVA-based materials are used in pharmaceutical and in biomedical fields as drug carriers and are also applied in tissue engineering science [56,57,58,59,60,61,62,63]. Additionally, PVAs with their crystalline structure consist of H-atoms interlinked between the hydroxyl group, and these hydrogen atoms could be interlinked [64]. The application of transdermal patches is being used as a component of the biomedical system because of the desirable PVA properties, such as water solubility and biodegradability. PVA cross-linked microspheres are used in oral precision relief systems [42,65].

Hydrophilicity and processing characteristics allow this polymer to be mixed with other natural and artificial polymers [38,66]. PVA composites in hydrogel form have been used extensively in the medical field because of their biocompatibility, and are a well-known polymer gel with several applications, such as in organ replacement, drug delivery devices, and wound management [52,53,67,68,69,70]. From this point of view, encapsulating measurement nanoparticle (MNP) in PVA is a challenging and promising topic. PVA’s good structure and biocompatibility with MNP, along with the cost of material, allow its biomedical and pharmaceutical applications [71,72]. PVA is a polymer that acts as a protective agent with formations in water solution and abundant OH groups; it also tends to absorb metal ions and form complex products [71,73]. Several research groups have investigated the application of nanotechnology in PVA, and they have reported their hydrogel preparation based on organically modified montmorillonite and studied their potential use as the main wound dressing devices *in vitro* [52,74] and *in vivo* [75] environments. 

Biocompatibility of HNTs was improved by wrapping amylase on the surface. Basically, the utilization of the amylase-HNTs for applications in absorbing metal ions or dyes can be regarded as absorbents in biomedical aspects and also as biological nanoreactors and nanofillers in biopolymer matrix. In addition, in hydrocarbon processing and catalytic conversions, HNTs were used for storing molecular hydrogen. In the environmental sector, HNTs present several biological and non-biological uses, such as a diuretic drug transportation to remove hazardous species [76].

### 6.2. General Applications of PVA and HNTs

Addition of HNTs was intended to make PVA water-insoluble and hence more useful in commercial applications. Examination of the composites indicated that HNTs were uniformly dispersed in both PVA as well as crosslinked PVA. Excellent mechanical properties of the PVA–HNT nanocomposites were achieved. These nanocomposites are intended to be composted at the end of their life rather than end up in landfills like most traditional petroleum-based non-biodegradable plastics [9]. PVA is used in many industries, such as textile, paper industry, and food packaging industry [29,54] because of its high chemical and thermal stability, and low manufacturing cost [77]. It is also a popular water-soluble polymer and has high strength and high optical transparency in water. Because of these desirable properties, PVA is used in packaging and as optical polarizer [43]. This polymer is also applicable in other industries, including polymer recycling, food packaging, binding and coating, and adhesives. PVA is a type of biodegradable resin even if it is an artificial polymer derived from petroleum [78]. The extensive use of PVA is observed in different fields of science and technology, such as coating and finishing agents, emulsifiers, wood, and leather [79]. PVA is an excellent source for fiber, polymer, textile, surface, organic [80].

## 7. Fabrication Approaches for PVA-HNTs Composites

HNTs can be mixed with most plastics using traditional equipment, such as the twin screw extruder and the two roll mill. The selection of fabrication methods has mainly focused on improvement of nanotube dispersion in the polymer matrix and on the enhancement of interfacial interactions. The common processing methods used for PVA-HNTs composites are presented in the following sections.

### 7.1. Dispersion Processing

**Figure 5:**
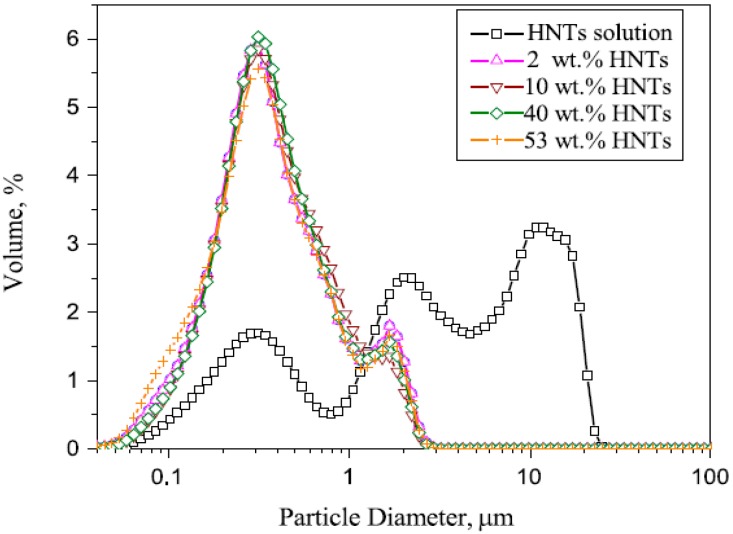
The particle size and distribution of HNTs in mixture of PVA-HNTs [3].

Dispersion mixing is the most commonly used method for fabricating PVA-HNT composites because it is easily performed and suitable for a small sample size. This process is realized by dispersing the nanotubes and PVA in a suitable solvent (*i.e.*, water and organic solvent) with vigorous stirring followed by ultrasonic treatment. Finally, the PVA-HNTs composites are obtained as film or hydrogels after casting [74]. The incorporation of HNTs significantly reduces the level of decomposition activity of the PVA backbone, thereby increasing its efficiency. The effect of low concentration of HNTs shows more significant effect compared with the effect of high concentration on the thermal decomposition of the composite films when the aggregation process is involved in high HNT concentration. Surface properties of the fillers determine the interaction between the fillers and PVA, which are of primary importance in filler selection criteria. Figure 5, adapted from [3], shows the particle size and distribution of HNTs in different PVA–HNT mixtures 

Considering the desirable features and solubility in water, HNTs are used as the carrier agent of a mixture in a mixture containing polymers for nanoparticles. As an example, PVA–HNT films have considerable mechanical and physical properties and can be processed through the selection of suitable PVA–HNT mixtures [81]. PVA–HNT films can be made by crosslinking with glutaraldehyde in an aqueous mixture and casting on glass substrates. PVA has remarkably high transparency, which indicates the homogenous distribution of HNT within the composite films [82].

### 7.2. Electrospinning

Electrospinning is a simple and versatile fiber synthesis technique in which a high-voltage electric field is applied to a stream of polymer solution, thereby forming continuous micro/nanofibers. Electrospun fibers create a fabric network with high porosity, very small pore size, and very large surface-to-volume ratio. Therefore, these materials could be used for many biomedical applications, such as drug delivery, artificial organs, wound dressing, and medical prostheses. The polymers used for electrospinning with HNTs range from polylactide (PLA) to PVA. Biodegradable PLA and PVA have good electrospinning properties, leading to their application in many areas. Dichloromethane or chloroform was employed to dissolve the PLA with dimethyl formamide to enhance the electric conductivity. Water was used as a solvent for PVA. Mixing the HNTs with the polymer solution before spinning could generate composite nanofibers [74]. Non-woven mats containing electrospun fibers with high porosity and large specific surface area provide a potential way of manufacturing high-performance fiber-reinforced polymer composites through polymer solution impregnation. Recent experiments have demonstrated that electrospun fibers are effective in reinforcing rubber films and enhancing the strength and stiffness of the epoxy matrix. The mechanical performance of the electrospun fiber reinforced polymer composites is known to critically depend on the interface adhesion between the fibers and the matrix [83].

## 8. Characterization of PVA–HNT Composites

In this section we discuss the preparation, characterization, water absorption capacity, thermal stability and flame retardant behavior of PVA-HNTs nanocomposites with special reference to filler loading.

### 8.1. Morphological Studies of PVA–HNT Nanocomposites

**Figure 6:**
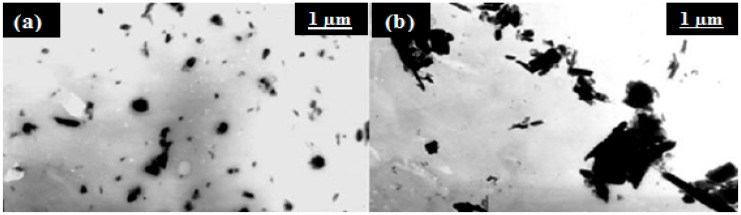
TEM photos of PVA*-*HNT nanocomposite films (2 wt % HNTs) prepared by: (**a**) coagulation (**b**) casting [3].

Figure 6 shows the physical aspect of nanocomposite films of PVA-HNT with 2 wt % HNT loading. Two methods were used to prepare the nanocomposite films. Moreover, the well dispersed HNTs in the mixture prepared by coagulation method separate from the solidified PVA during the precipitation process and cannot reaggregate [3].

**Figure 7:**
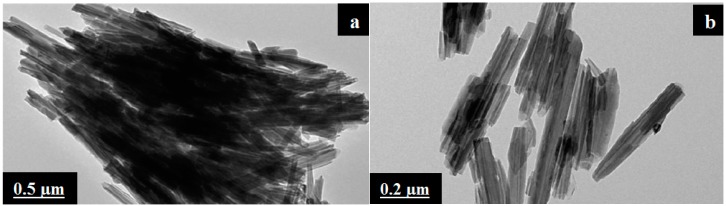
TEM images of HNTs: (**a**) untreated and (**b**) after acid treatment [7].

**Figure 8:**
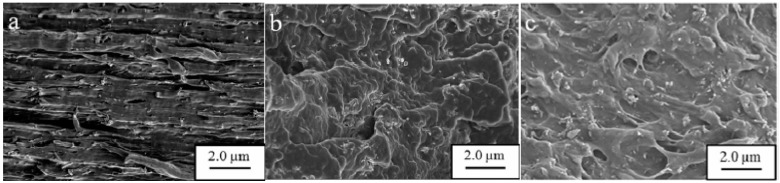
SEM images of fractured surfaces of PVA-HNT films after different H_2_SO_4_ treatment times (8 wt % HNT loading); (**a**) untreated; (**b**) H_2_SO_4_ treatment for 1 h and (**c**) H_2_SO_4_ treatment for 8 h [66].

Sulfuric acid treatment was performed for only 1 h, particularly when a high Young’s modulus is desired. Longer sulfuric acid treatment would decrease its tensile strength. Sulfuric acid dissolves Al^3+^ ions, thereby resulting in HNTs that consist of amorphous silica. Thus, the reduction in the crystalline structure of HNTs was first considered. Figure 7 shows TEM images of untreated HNTs (Figure 7a) and 8 h-treated HNTs (Figure 7b). Untreated HNTs have a clear hollow tubular structure, whereas the tubular profile is difficult to identify for sulfuric acid-treated HNTs [43].

The fractured surfaces of untreated, H_2_SO_4_ treated for 1 h, and H_2_SO_4_ treated for 8 h PVA-HNT films are shown in Figure 8. Figure 8a shows traces of resin elongation originating from a slip caused by poor adhesion of PVA and HNTs, but Figure 8b exhibits no slippage, and the HNTs are well distributed in PVA. Nonetheless, traces of resin elongation, as well as untreated HNTs, are shown in Figure 8c. Thus, long treatment times with H_2_SO_4_ may negatively affect the interfacial adhesion between PVA and HNTs [43].

### 8.2. Thermal Properties of PVA-HNT Nanocomposites

**Figure 9:**
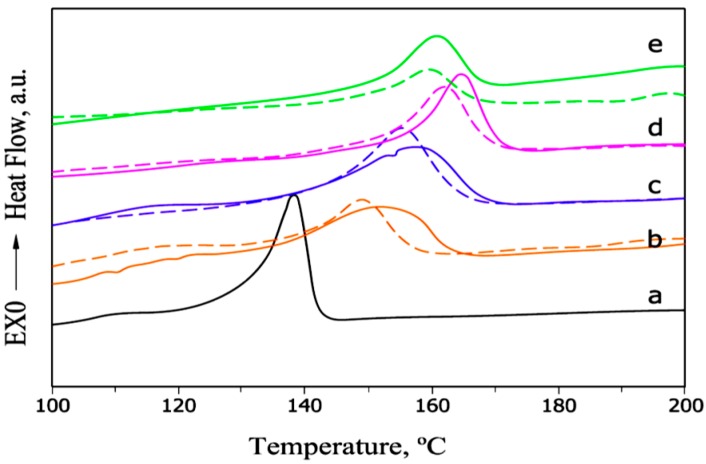
DSC cooling thermograms of neat PVA and PVA-HNT composite films with different HNT contents: (**a**) 0 wt % (neat PVA); (**b**) 2 wt %; (**c**) 10 wt %; (**d**) 40 wt %; (**e**) 53 wt % [3].

PVA is a partially crystalline-structured polymer with physical and chemical properties, such as interchain and intrachain polymer interactions, because of the H-bonding in between hydroxyl groups. The introduction of nanosized HNTs particles with –OH groups changes the intermolecular and intramolecular interaction between the polymer chains, which in turn changes the composition of the physical structure and the crystallization behavior. Thus, the properties of PVA films change. Figure 9 shows a DSC curve of neat PVA and PVA-HNT composites in cooling mode, thereby illustrating that the crystalline PVA peak structure shifts to high temperature with increasing HNT contents and the crystallization of neat PVA is more persistent [3].

**Figure 10:**
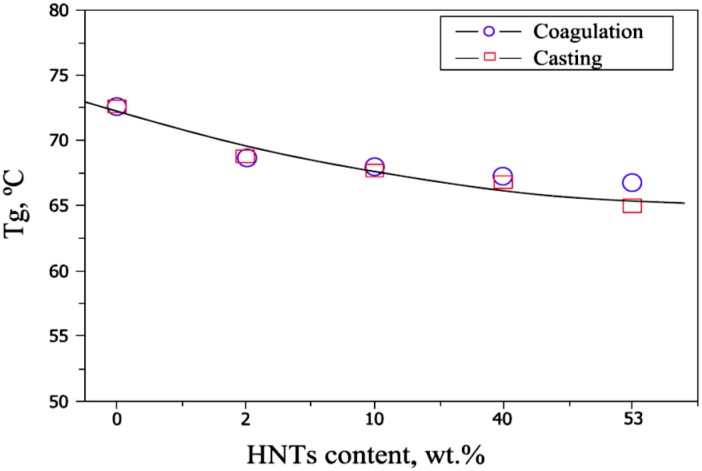
T_g_ curves of PVA-HNT composites with different HNT contents [3].

Figure 10 shows the glass transition temperature (T_g_) of different composites. A gradual decrease in T_g_ with increased HNT contents in the polymer composition is observed, and the preparation method has no effect on the T_g_ of the resulting PVA-HNT composite film. The effect of measurements of nanoparticles on T_g_ of PVA composites is the opposite [3].

**Figure 11:**
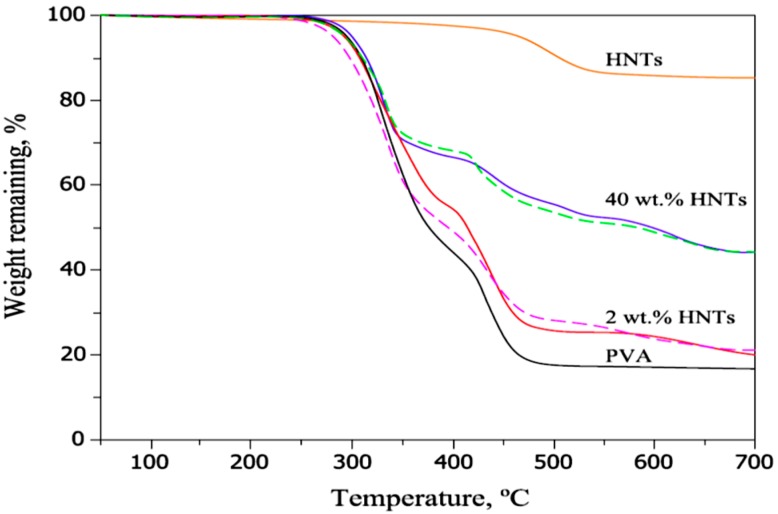
TGA curves of PVA, HNTs and PVA-HNT composites [3].

The TGA curves of PVA, HNTs, and PVA-HNT composites are shown in Figure 11. The neat PVA curve is represented by two weight loss steps. The weight loss of composites near 350 °C and 450 °C is due to the decomposition process of crosslinked chains and the basic units of PVA, respectively [3].

The small difference between T_m_ and the decomposition temperature of PVA causes difficulty in production of the film. The polymer’s use in the food industry is limited to packaging applications because of the rising cost of the production process. To improve simple packaging film formation, the PVA thermal properties are enhanced [79]. The versatility of HNTs in producing varieties of enhanced PVA composites and the simple production of PVA-HNT composites and the enhanced thermal properties consolidate its novelty. Table 3 presents a list of HNT-based PVA composites with the ranges of HNT loading and the intended/achieved target thermal properties for each composition. 

**Table 3:** HNT-based PVA composites with the range of HNTs loading and the intended/achieved target thermal properties for each composition.

HNTs (wt %)	Form	Property Improvements (Percentage %)				Ref.
		T_c_ (°C)	T_g_ (°C)	T_m_ (°C)	TGA (°C)	
0, 2, 10, 40 & 53	Film	-	12.3	-	28.6	[3]
0 & 10	Film	12	-	1.8	-	[6,7]
0, 3.75, 7.5 & 15	Modification	-	-	-	14.3	[7,19]
0, 5, 10 & 20	Reinforce	-	12.8	5.2	30	[9]
0, 1, 2.5 & 5	Reinforce	2.6	3.9	0.5	8.8	[47]
0, 2, 5, 7.5 & 10	Film	-	-	0.2	-	[53]
0, 0.25, 0.5, 1, 3 & 5	Film	-	-	-	14.5	[72]

### 8.3. Mechanical Properties of PVA-HNT Nanocomposites

The tensile strength of PVA-untreated halloysites film was reduced, but the Young’s modulus increased compared with neat PVA film. Sulfuric acid treatment was conducted on PVA to improve its tendency and to create film composites. This treatment is applied on the HNTs for the improvement of interfacial adhesion. Treated HNTs improved the tensile strength of PVA-HNTs films when the nanotubes were subjected to sulfuric acid treatment for a short time, less than 2 h. Polymer composites and nanoclay particles are usually called polymer or clay nanocomposites. These small amounts of filler greatly increased the strength because of the magnitude of the specific area [20,43] of the nanoclay particles or composites. Blending with HNTs induces changes in the nano-physiology and causes changes in the chemical structure of PVA films. The incorporation of HNTs enhances the mechanical properties of PVA [82]. 

**Figure 12:**
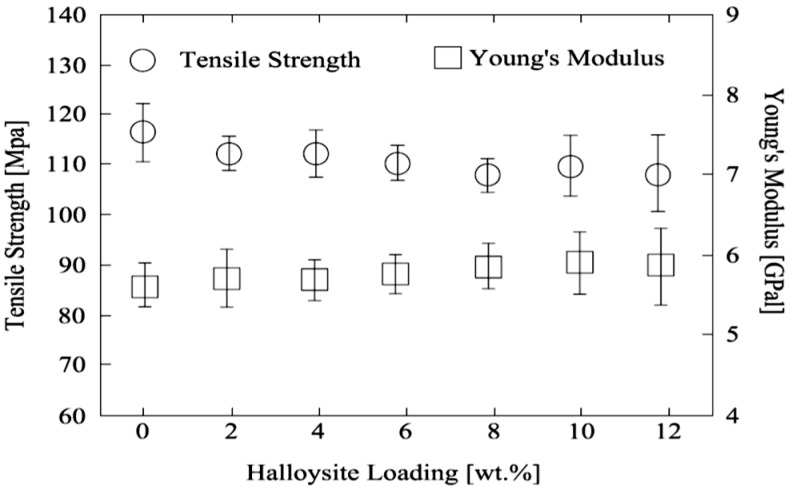
Tensile strength and Young’s modulus of PVA-HNT films with different amounts of HNTs [66].

PVA has high tensile strength and is soluble in water. Figure 12 describes the relationship between tensile strength and Young’s modulus of PVA–untreated HNT films, with various HNT loadings. As the contents of HNTs increase, the tensile strength decreases with less ratio, and the Young’s modulus is greater. Only the lack of adhesion capacity between the polymer matrix and fillers reduced the strength [66]. The versatility of HNTs for producing varieties of enhanced PVA composites, and the production of PVA-HNTs nanocomposites and the enhanced mechanical properties consolidating their novelty. Table 4 presents a list of HNT-based PVA composites with the range of HNT loadings and the intended/achieved target mechanical properties for each composition.

**Table 4:** HNT-based PVA composites with the range of HNTs loading and the intended/achieved target mechanical properties for each composition.

HNTs (wt %)	Form	Property Improvements (Percentage %)			Ref.
		Tensile Strength	Young’s Modulus	Elong. at Break (mm/mm)	
0, 3.75, 7.5 & 15	Modification	13.6	-	-	[7,19]
0, 5, 10 & 20	Reinforce	22.7	407.1	-	[9]
0, 2, 5, 7.5 & 10	Film	81.8	-	-	[53]
0, 2, 4, 6, 8, 10 & 12	Film	6.4	80.2	-	[66]
0, 0.25, 0.5, 1, 3 & 5	Film	20	94.9	82.9	[72]

## 9. Conclusions

PVA is an artificial polymer that has been used in the medical and other fields for the last 30 years. This polymer has been studied widely based on clinical and nonclinical research. PVA-HNT nanocomposites can be used in general clinical operations such as cartilage replacement or cartilage transplantation because they have the advantage of being readily available compared with cartilage transplantation. Disease transmission concerns and limited availability characterize cartilage transplantation. Moreover, PVA-HNT nanocomposites can be used in eye contact lenses, eye drops, and drug delivery systems to target tissue with abnormal growth rates. In summary, we have reviewed PVA and PVA–HNT nanocomposite applications due to their biocompatibility, non-toxicity, noncarcinogenicity, smoothness, and flexibility in potential applications in bone tissue engineering and drug delivery systems.
